# Reports of Societies

**Published:** 1875-09

**Authors:** 


### Reports of Societies.

### CHICAGO SOCIETY OF PHYSICIANS AND SURGEONS.

*Regular Meeting, July* 26, 1875.

(Dr. Owens, Reporter *pro tem.*)

Thomas Bevan, M.D., President, in the chair.

### HISTORY OF A CASE OF IDIOPATHIC SUBACUTE NEURITIS, AFFECTING THE CRURAL, OBTURATOR AND SCIATIC NERVES.

By Henry M. Bannister, M.D.

The case described was that of a young man, 23 years old, who, after exposure to cold, suffered from violent pains in the left groin, coming on suddenly and continuing several hours. After their disappearance, tenderness and soreness, with a considerable degree of weakness, still remained in the limb, disabling him from standing at his occupation, that of a painter. This condition lasted several weeks, when a sudden, rather rough handling brought on the pain a second time, and greatly increased the weakness, rendering locomotion difficult. After several weeks of this state of things, he left his work and returned to his home at Evanston, Ill., and put himself under the care of a physician who treated him at first for sciatica. At this time, possibly owing to some feature of the treatment, he suffered an aggravation of all his symptoms. The neuralgic pains increased in frequency and violence, the reflex excitability was greatly exalted, and for several days he was unable to lie down or even straighten his leg. The symptoms as described at this time seemed to resemble those of the second stage of coxalgia, and on this diagnosis he was finally put to bed and extension applied to the limb, with the result of giving partial relief.

At the time when first seen by Dr. Jewell and myself, he had dismissed his physicians and had been under his own care solely for three weeks. His general condition was fair, but he was unable to move his left leg to any great extent, or to lie on either side, tenderness existed over the adductor muscles, along the course of the sciatic and in the sciatic notch, and to a certain extent all around the hip joint, the tissues in that region being somewhat swollen and tense. The crural nerve seemed less tender. The patient had himself removed the extension apparatus some days before, and now both extension and compression of the joint caused pain, sometimes referred to the knee and sometimes to the groin.

The diagnosis of neuritis being made, the treatment prescribed was absolute rest for the limb, the galvanic current, small blisters around the joint, and occasional friction of some of the affected muscles. The patient's circumstances prevented any costly appliances that might have been useful, and considerably embarrassed the treatment. After three weeks of this treatment, a considerable improvement was manifest. The swelling had largely gone down, the tenderness over the sciatic and around the hip joint had disappeared to a very great extent, and power of motion had largely returned to the limb—so much so, that it seemed advisable to use mechanical means for enforcing absolute rest of the part, which was before insured by its weakness.

The case is yet under observation.

Dr. Bannister considered the case as one of idiopathic subacute neuritis, affecting the obturator, crural and sciatic nerves probably—the first certainly. It is, in many respects, a typical case of this disease; in others, it differs from what is usually described. Its interest is in the comparative rarity of the affection in its well developed form, and although not uncommon it is not an everyday disorder. It probably happens much oftener than it is recognized. Some authors place it amongst the rarest of affections of nerves, *i. e.*, in its idiopathic, non-traumatic form, like the case described, but in this, he thinks, they are in error.

Weir Mitchell's book on nerve injuries gives, perhaps, more information in regard to the subject than any other work in our language. The latter believes fully in it, as a factor in the pathology of many cases of disorder of the nerves. With the exception of papers concerning a similar affection of the optic nerve, and a few on disorders of nerves as productive of certain skin affections, the literature is quite insignificant.

In other languages the same is the case to a very great degree, but foreign text books are, many of them, fuller in their treatment of the subject—Erb, for instance, in his work on the principal nervous affections, in the 12th vol. of Ziemssen's Encyclopædia. The case had been variously diagnosed—hip-joint disease, neuralgia, rheumatism.

Dr. Bannister saw no reason for doubt as to the diagnosis of neuritis. The lack of sensory symptoms, and the rapid and variable character of the paralytic symptoms, are noteworthy. The variable character of the tenderness, now in one place more than another, and then again changing, but nevertheless always persisting to a greater or less degree, is worthy of mention.

Perhaps the best reason for the disproportionate ignorance of this subject, is the fact, that neuritis seldom is fatal when confined to the peripheral nerves, and the central lesions to which it gives rise, are alone noticed in the autopsy. It would require a very careful clinical history and analysis of any case to determine whether peripheral nerve changes are primary or secondary. There is considerable reason to think, that a morbid process starting in the nerves, may rapidly involve the cord and medulla, and cause death.

The case was discussed by Drs. Hyde, Merriman, Peck, and others.

### CHICAGO SOCIETY OF PHYSICIANS AND SURGEONS.

*Regular Meeting, Aug.* 9, 1875.

(Reported by E. WARREN SAWYER, M.D.)

The Society met at the Grand Pacific Hotel. The President, Dr. Bevan, in the chair.

Prof. Freer, the essayist of the evening, read an interesting and extended paper on the subject of transfusion, which will appear, *in extenso*, in the Transactions of the State Medical Society.

The first part of the paper was given to the history of transfusion to the present time.

Two distinct methods of transfusing are known to-day; first, the mediate. The reader exhibited the various instruments which had been used for transfusing mediately; the simple hydrocele syringe he had used more than once successfully, as had also Dr. Hunt. Especial attention was called to an ingenious apparatus suggested by Dr. F. L. Wadsworth, which consisted of a graduated Wolf bottle; in the central opening a perforated, tightly fitting cork was placed, which gave passage to a thermometer, by means of which the temperature of the blood to be used was always known. Through one lateral opening in the bottle, a tube was passed through a tightly fitting cork, one end of which was armed with a blunt-pointed canula which is to be introduced into a vein of the recipient; the other end of the tube reaches nearly to the bottom of the bottle. Through the other lateral opening in the bottle, a tube is passed through a tight cork, one end of which extends a little way into the bottle, the other end communicates with a common Davidson's syringe. The bottle is now to be partly submerged in a vessel of warm water, the temperature of which can be regulated by pouring into this vessel hot water from time to time. By working the bulb of the syringe, the atmospheric pressure within the bottle is increased, and the blood can thus be forced into the receiver's veins.

Prof. Freer gives preference, above all other instruments, to the simple pipette suggested by McDonald, of Dublin.

By the immediate method, the blood is passed directly from the veins of the donor to the veins of the receiver, through a simple tube, with no other *vis a tergo* than the cardiac impulsion of the donor. This method is confined almost entirely to Germany ; in fact, the alarming symptoms which it causes, precludes its use in any other country.

A kind of bogus immediate method of transfusion had been conceived by Aveling, whose instrument is a valveless Davidson's syringe, which is placed in communication with the vein of the donor and receiver. By working the bulb of the instrument, and pinching the rubber tube alternately on the proximal and distal side of the bulb, the blood is aspirated from the vein of one and forced into the other's vein ; the bulb being a substitute of the ventricle of the blood donor.

This is the most dangerous of all modes of transfusion, for it is almost impossible to avoid the coagulation of the blood, and the escape of small clots into the vessels of the receiver.

The virtues of these two methods, of mediate and immediate transfusion, are not comparable. In the one, blood must be used which will coagulate, under some circumstances, despite the greatest rapidity of operation and the utmost circumspection. In the other, blood is used which cannot coagulate ; thus allowing the operator to enjoy the freest leisure at the same time that he entertains no fear that the person will be destroyed by the operation. In fact, "*you cannot injure your patient, if you would*," by using the defibrinated blood.

The reader preferred a whisk of broom corn, made clean by boiling, to all other means of whipping and defibrinating the blood.

Human blood should be used in human transfusion, for recent experiments in animal transfusion have shown that the blood serum of one species is inimical to the blood corpuscles of another species.

The physiological import of the operation is worthy of the most extended consideration. Prof. Freer reiterated his convictions that there is nothing mysterious connected with the effect of transfusion, as there is in the action of a drug ; it should be regarded as a means of nutrition, simply, in which a food is supplied in a form most perfectly digested and prepared, and ready to be assimilated by the various tissues. He is satisfied that fibrine is not essential to the constitution of this perfect food, and further, because its presence adds greatly to the real danger in the operation, it not only *can* be removed without detracting from the virtue of the food, but it *should* be removed in order that the risks of the operation may be averted.

The great reason why so little has been realized from transfusion, is because the nutritive end of the operation is not considered ; and because the supplying of this pabulum is not repeated, or, if repeated, an interval is observed, during which the patient dies from inanition.

Prof. Freer alluded to a series of experiments which he had performed,* which showed that defibrinated blood could be kept for a long time without destroying its vitality.

Many instances of transfusion were spoken of which had been performed in this country and abroad. The paper was quite generally discussed by the gentlemen present.

Dr. Bartlett addressed the Society on the subject of determining the amount of urea contained in urine by Apjohn's method. His subject was fully illustrated with chemical apparatus, and by the examination of a specimen of urine. The speaker called attention to the importance of reading the index after the tube had been made to communicate with the retort, and the disparity between the level of the fluid in the containing tube and the inner

* CHICAGO MEDICAL JOURNAL, 1875, No. 7, page 518.

tube be noted, before the urine is mixed with the test solution. This disparity is to be subtracted from the index after the generation of nitrogen has ceased.

Two ingenious and valuable details in this mode of analysis have been suggested by Mr. N. Gray Bartlett. First, the submerging of the retort in a jar of water, to cool the contents of the retort after the generation of the nitrogen. Second, the pouring of the urine into the retort, and having the test solution in the bottle, which is to be mixed with the urine, instead of the reverse order; with this advantage, viz., the test solution may be measured out in little charges which can be kept on hand by the analyst.

Dr. Sawyer reported the case of a gentleman who was the victim of periodical blood-losing by venesection.

Dr. Bartlett exhibited a modification of Neugebauer's speculum, which possessed the advantages over the original, in that it could be used without moving the patient from the middle of the bed, and without an assistant to hold the handles.

---

Poisoning by Strawberries.—Dr. Garnier relates a case of fatal poisoning by strawberries (in the *Lyon Médical* for June 20th), occurring in a strong, healthy girl, 15 years of age. Three hours after eating a cup of berries, "he found her in a dying state, comatose, with complete prostration, livid countenance, and a very small, intermitting pulse." All remedial measures signally failed. The writer has seen several similar cases, and has saved four by emetics, but he has lost more than he has saved. Enormous meteorism is speedily produced, and in consequence thereof, in Dr. G.'s opinion, after two hours, emetics are of no avail; instead of emetics, then, ammonia should be given.

